# Chemical Properties of Air Pollutants and Cause-Specific Hospital Admissions among the Elderly in Atlanta, Georgia

**DOI:** 10.1289/ehp.1002646

**Published:** 2011-06-27

**Authors:** Helen H. Suh, Antonella Zanobetti, Joel Schwartz, Brent A. Coull

**Affiliations:** 1 Environmental Health Program, NORC at the University of Chicago, Boston, Massachusetts, USA; 2 Department of Environmental Health, and; 3 Department of Biostatistics, Harvard School of Public Health, Boston, Massachusetts, USA

**Keywords:** air pollution, chemical properties, hospital admissions, multipollutant analysis, transition metals

## Abstract

Background: Health risks differ by fine particle (aerodynamic diameter ≤ 2.5 μm) component, although with substantial variability. Traditional methods to assess component-specific risks are limited, suggesting the need for alternative methods.

Objectives: We examined whether the odds of daily hospital admissions differ by pollutant chemical properties.

Methods: We categorized pollutants by chemical properties and examined their impacts on the odds of daily hospital admissions among Medicare recipients > 64 years of age in counties in Atlanta, Georgia, for 1998–2006. We analyzed data in two stages. In the first stage we applied a case-crossover analysis to simultaneously estimate effects of 65 pollutants measured in the Aerosol Research and Inhalation Epidemiology Study on cause-specific hospital admissions, controlling for temperature and ozone. In the second stage, we regressed pollutant-specific slopes from the first stage on pollutant properties. We calculated uncertainty estimates using a bootstrap procedure. We repeated the two-stage analyses using coefficients from first-stage models that included single pollutants plus ozone and meteorological variables only. We based our primary analyses on exposures on day of admission.

Results: We found that 24-hr transition metals and alkanes were associated with increased odds [0.26%; 95% confidence interval (CI), 0.02–0.48; and 0.37%; 95% CI, 0.04–0.72, respectively] of hospital admissions for cardiovascular disease (CVD). Transition metals were significantly associated with increased hospital admissions for ischemic heart disease, congestive heart failure, and atrial fibrillation. Increased respiratory-related hospital admissions were significantly associated with alkanes. Aromatics and microcrystalline oxides were significantly associated with decreased CVD- and respiratory-related hospital admissions.

Conclusions: The two-stage approach showed transition metals to be consistently associated with increased odds of CVD-related hospital admissions.

Air pollution has consistently been associated with increased hospital admissions in cities throughout the United States and the world. Recent studies suggest that the increase in hospital admissions is due primarily to fine particles [particulate matter ≤ 2.5 μm in aerodynamic diameter (PM_2.5_)] (Klemm et al. 2000; Schwartz et al. 1996). Major PM_2.5_ components vary by region and by season, but typically include ammonium sulfate and nitrate, elemental carbon (EC), carbonaceous species, carbonates, metals, and water. Despite considerable research, the relative toxicity of different constituents of PM_2.5_ remains unclear but likely varies.

Results from epidemiological and toxicological studies support this theory. Multicity studies of acute PM show that associations between ambient particles and hospital admissions vary by city (Dominici et al. 2006; Janssen et al. 2002; Zanobetti et al. 2009), with this variation attributed in part to particle composition differences. Zanobetti et al. (2009), for example, used a hierarchical approach that controlled for seasonal temperature to determine how the association between daily PM_2.5_ mass and hospitalizations was modified by PM_2.5_ composition in 25 U.S. communities. They found that effect estimates for PM_2.5_ total mass and cardiovascular hospital admissions were higher on days with higher PM_2.5_ content of bromine, chromium, nickel, or sodium ion. In a study that did not control for seasonal temperature, Bell et al. (2009) identified a different set of modifiers for associations between hospitalizations and PM_2.5_, including EC, vanadium, and nickel. In Atlanta, Georgia (USA), analyses of air quality data from the Aerosol Research and Inhalation Epidemiology Study (ARIES) showed significant associations between cardiovascular disease (CVD) emergency department visits and nitrogen dioxide (NO_2_), carbon monoxide (CO), EC, organic carbon, and oxygenated hydrocarbons (Metzger et al. 2004; Tolbert et al. 2007) and between cardiopulmonary emergency department visits and pollutant groups consistent with motor vehicles, metal processing, biomass burning, and wood smoke (Sarnat et al. 2008). For respiratory visits, associations were found with ozone, NO_2_, CO, and inhalable particles (PM_10_) (Peel et al. 2005; Tolbert et al. 2007). Associations of these particle components and sources with emergency department visits varied by visits related to cardiovascular or respiratory conditions, suggestive of different component-mediated biological pathways.

These studies provide important evidence that health risks differ by pollutant. However, the pollutants posing the greatest concern have generally varied by study and health outcome, even when conducted within the same city, raising concerns regarding the reliance on many single-pollutant models to examine air pollution impacts. To address these concerns, studies have examined the risks associated with multiple pollutants using source apportionment approaches (Sarnat et al. 2008; Thurston et al. 2005). These approaches group pollutants according to how their daily concentrations covary, presumably because they are generated from common sources. This approach is advantageous if the responsible pollutants are largely from one source. However, it also may group elements with high toxicity and low toxicity together, thereby masking health effects that may be specific to individual elements. Consequently, source apportionment approaches may be limited in their ability to identify individual toxic pollutants. Further, results are often not generalizable to other cities, given that each city may have a different source profile.

Methods based on the chemical properties or structures of pollutants offer an alternative approach to assessing health impacts from multiple pollutants. Grouping pollutants by chemical properties may be advantageous if the properties are related to toxicity and if multiple measured pollutants have the same toxic effect. Chemical-property–based approaches to assess toxicity or biological activity have long been used to design pharmaceuticals (Gozalbes et al. 2002; Hajduk and Greer 2007) and have been adapted by the U.S. Environmental Protection Agency (EPA) and others to predict the toxicity of new chemicals, to assess potential risks, and to make regulatory decisions (Cronin et al. 2003; Eriksson et al. 2003). Several Chemical-property–based methods have been developed, including structure–activity relationships, quantitative structure–activity relationships, nearest analogue analysis, and chemical class analogy (Cronin et al. 2003; Eriksson et al. 2003; Karcher and DeVillers 1990). However, to our knowledge, to date these methods have not been adapted in epidemiological air pollutants studies.

In this article, we assess whether a pollutant’s chemical properties are related to adverse health impacts, using a hierarchical two-stage regression (Greenland 1993, 2000; Witte and Greenland 1996) that examines the relationship of chemical properties and cause-specific hospital admissions. Specifically, we tested the hypothesis that health effects of air pollutants differ by pollutant chemical properties, consistent with variation in toxicity and the biological pathways mediated by different pollutants. We compared our findings with those from traditional single-pollutant analyses.

## Materials and Methods

Our study was conducted under a protocol approved by the Harvard School of Public Health Human Subjects Committee.

*Data sources.* Cause-specific hospital admissions data. We obtained information on hospital admissions for 1998 through 2006 for Medicare recipients > 64 years of age from the Centers for Medicare and Medicaid Services, which provide reimbursement of inpatient hospital costs for most U.S. citizens and permanent residents. Each record contains information on the date of hospital admission, age, sex, self-reported race, place of residence, and primary and secondary diagnoses. Using codes from the *International Classification of Diseases, Ninth Revision, Clinical Modification* (ICD-9-CM; National Center for Health Statistics and Centers for Medicare and Medicaid Services 2005), we considered hospital admissions for all CVD conditions (codes 390–429), for all respiratory outcomes (codes 460–519), and for specific CVD or respiratory conditions: congestive heart failure (CHF; code 428), myocardial infarction (MI; code 410), ischemic heart disease (IHD; codes 410–414), atrial fibrillation (code 427.31]), chronic obstructive pulmonary disease (COPD; codes 490–492, 494–496), pneumonia (codes 480–487), and acute respiratory infection (ARI; codes 460–466). Outcomes were selected based on findings from previous air pollution health studies (Bell et al. 2009; Dominici et al. 2006; Rich et al. 2006; Zanobetti et al. 2009). In a study of implantable cardioverter defibrillators, for example, episodes of paroxysmal atrial fibrillation episodes were positively associated with increased ozone and, to a lesser extent, PM_2.5_, NO_2_, and black carbon in the hour before the arrhythmia (Rich et al. 2006). We included only those admissions that occurred through the emergency department, because scheduled, nonemergent admissions, such as those for elective surgery, are likely not related to air pollutant exposures. Data for all hospitals within Cobb, DeKalb, Gwinnett, and Fulton counties, which encompass metropolitan Atlanta, were included in our analyses.

Pollution data. We obtained daily data for 122 unique pollutants and meteorological variables measured at the Jefferson Street stationary ambient monitoring site as part of the ARIES study (Hansen et al. 2006). PM_2.5_ and its component concentrations were measured using 24-hr integrated Federal Reference Methods (FRM; Hansen et al. 2006). We filled in missing 2004–2006 gaseous pollutant data by calculating 24-hr averages (midnight to midnight) of hourly ambient sulfur dioxide, ozone, NO_2_, and CO concentrations from U.S. EPA monitoring sites in Fulton County. Samples with values below the limit of detection (LOD) were set to half the LOD in the ARIES data set.

*Data analysis.* We estimated average effects of interquartile range (IQR) increases in pollutants with the same chemical properties on the odds of hospital admissions using a two-stage approach that has been used to estimate associations between a phenotype and a large number of single-nucleotide polymorphisms (Conti and Gauderman 2004; Greenland 1993, 2000; Thomas et al. 2007; Witte and Greenland 1996). In the first stage, we performed a case-crossover analysis for each outcome to obtain regression coefficients standardized to IQR increases in each pollutant. The second stage used a metaregression approach that regressed the first-stage coefficients against the identified pollutant-property categories.

Data summaries. We performed univariate explorations of all variables using histograms and statistical summaries. Pollutants were included in further analyses if they were measured (and estimated as for the gases) for > 75% of the study period, they had an IQR-to-median ratio > 0.30, and 90% of their measured values exceeded their LOD. We used the IQR-to-median ratio instead of the coefficient of variation as a selection metric because it is less influenced by extreme values. Pollutants with IQR-to-median values < 0.30 were excluded from the analyses because these pollutants lacked sufficient variation to estimate effects with reasonable power.

Pollutant properties. Pollutants were classified by their chemical properties based on scientific literature (Haynes 2011). Pollutants generally fell within easily definable major chemical property groups (e.g., aromatics, transition metals). As appropriate, we classified each pollutant according to multiple chemical properties, for example, classifying benzene as both an “aromatic” and “combustible” pollutant. Although alkanes and aromatics are inert pollutants, we excluded alkane and aromatic pollutants from the inert classification to maximize differences between chemical property groups. Similarly, we excluded aldehydes from the polar category. Pollutants initially were classified according to 12 different chemical properties, but we limited analyses to nine chemical properties that included at least three pollutants (Table 1). Consequently, several pollutants, including ozone, were not included in any of the chemical property groups evaluated in the two-stage analysis.

**Table 1:** Pollutants by category.

Pollutant category	Pollutants
Inert (nonaromatic, nonalkane)	Cyclopentane, methylcyclopentane, methylcyclohexane, sulfur, EC, CO, NO_2_
Polar (nonaldehyde)	Acetic acid, 2-butanone, organic carbon
Aromatic	Benzene, toluene, ethylbenzene, *m*-xylene and *p*-xylene, *o*-xylene, *n*-propylbenzene, *p*-ethyltoluene, *m*-ethyltoluene, 1,3,5-trimethylbenzene, 1,2,4-trimethylbenzene and *sec*-butylbenzene
Aldehyde	Benzaldehyde, heptanal, hexanal, octanal
Alkane	2,2,4-Trimethylpentane, 2,2-dimethylbutane, 2,3,4-trimethylpentane, 2,3-dimethylbutane, propane, 2,3-dimethylpentane, 2,4-dimethylpentane, 2-methylheptane, 2-methylhexane, 2-methylpentane, 3-ethylhexane, 3-methylhexane, 3-methylpentane, ethane, isobutane, isopentane, *n*-butane, *n*-decane, *n*-heptane, *n*-hexane, *n*-nonane, *n*-octane, *n*-pentane
Acidic	Acetic acid, nitric acid, sulfur, sulfur dioxide
Combustible	Ethane, propane, isobutane, *n*-butane, *n*-decane, isopentane, *n*-pentane, 2,2-dimethylbutane, cyclopentane, 2,3-dimethylbutane, 2,4-dimethylpentane, 2-methylpentane, 3-methylpentane, *n*-hexane, methylcyclopentane, 2-methylhexane, 2,3-dimethylpentane, 3-methylhexane, 2,2,4-trimethylpentane, *n*-heptane, methylcyclohexane, 2,3,4-trimethylpentane, 2-methylheptane, 3-ethylhexane, *n*-octane, *n*-nonane, benzene, toluene, ethylbenzene, *m*-xylene and *p*-xylene, *n*-propylbenzene, *p*-ethyltoluene, *m*-ethyltoluene, 1,3,5-trimethylbenzene, 1,2,4-trimethylbenzene and *sec*-butylbenzene, 2-butanone, ethylene, acetylene, propene, isobutene, 1-butene, *o*-xylene
Transition metal oxide	Copper, manganese, zinc, titanium, iron oxide
Microcrystalline oxide	Arsenic, bromine, selenium, lead, silicon oxide

In the first stage, we applied a case-crossover analysis, using a time-stratified approach. For each subject, we chose control days within the same month of the same year that the admission occurred, leaving 2 days between each control day to minimize serial correlation (Schwartz 2004). Using control days close in time to the event limits confounding by slowly varying personal characteristics, because each subject serves as his or her own control, and prevents confounding due to seasonal variation in exposures (Bateson and Schwartz 2001). We ran conditional logistic regression models by cause-specific hospital admission, for all dates and stratified by season. Seasonal analyses were performed using stratified data for ease of computing; as a result, we did not assess whether differences in the odds of hospital admissions were statistically different by season. We instead noted the magnitude, direction, and significance of the odds of hospital admission by season. We included all eligible pollutants in each first-stage model, with concentrations scaled by their IQR, and controlled for same-day linear average temperature, 1- to 3-day averaged temperature, and dew point temperature, and ozone. We also controlled for ozone, given its *a*) observed association with adverse effects (Peel et al. 2005), *b*) weak to moderate correlations with other pollutants, and *c*) exclusion from pollutant properties included in our analyses. The main analyses estimated associations with 24-hr average pollutant exposures on the day of admission. For the two main outcomes (CVD or respiratory hospital admissions), we also estimated associations with 2-, 3-, and 4-day moving average exposures that included exposure on the day of admission.

In the second stage, we modeled the set of coefficient estimates from the first-stage model in a weighted least-squares regression for a continuous response. Let *J* denote the number of pollutants in the model, and let *P* denote the number of pollutant characteristic covariates upon which the first-stage regression coefficients are regressed. Then, for coefficient *b_j_* corresponding to pollutant *j*, *j* = 1, . . ., 65, the second-stage model is

*b_j_* = γ*u_j_* + δ*_j_* + ε*_j_*, [1]

where *u_j_* is the 1 × *P* vector of pollutant property indicators (0/1 dummy variables, where 1 indicates inclusion in the chemical property group); γ is the *P* × 1 vector of second-stage coefficients, with each individual coefficient representing the average log odds ratio associated with an IQR increase in the pollutants included in that chemical property group; δ*_j_* is the chemical-property–specific error with the vector of all such errors assumed to have a multivariate normal distribution with 0 mean and fixed variance equal to the estimated variance–covariance matrix obtained at the first stage of the model; and ε*_j_* an unknown normal random error. We reported results from the second stage as the average percent change in odds of hospital admissions per IQR change in pollutants with the relevant chemical property.

To obtain valid confidence intervals (CIs), we used a bootstrap procedure (Efron and Tibshirani 1993), which was conditioned on the total number of admissions (*n*) in the study. We resampled *n* subjects with replacement and refitted both the first- and second-stage models to the resampled data set. We repeated this procedure 500 times, with 95% bootstrap CIs calculated using the 2.5% and 97.5% percentiles of the resulting sample of coefficient estimates.

Because we allowed pollutants to be classified into multiple pollutant-property categories, we examined potential multicollinearity among the categories using the eigenvalues of the variance–covariance matrix of the second-stage regression coefficient estimates (γ). We identified redundant properties given other properties by inspecting the pairwise correlations in this matrix and removing properties that had multiple pairwise correlations with other properties > 0.50. This procedure resulted in the omission of three pollutant properties from the original list (alkenes, nonpolar, gas/particle). Because alkanes and aromatics had a pairwise correlation > 0.50 but were not correlated with other properties, it was not clear which of the two properties should be omitted. As a result, we created two final groups of chemical properties for analysis. Both groups included inert (excluding alkanes, aromatics), polar (excluding aldehydes), aldehyde, acidic, and combustible compounds; transition metals; and microcrystalline oxides. Group A included aromatic compounds, and group B included alkanes. We fitted separate second-stage models for group A and group B properties. In total, we included 65 (including ozone) of the 122 measured pollutants in subsequent analyses. Observations were restricted to the 729 days (271 winter, 458 summer) for which data for all 65 pollutants were available (22% of 3,285 total days during 1998–2006).

Sensitivity analysis. To assess the influence of a multiple-pollutant model on first-stage coefficients, we also ran the first stage as a case-crossover analysis using models with single pollutants plus ozone (as opposed to multiple pollutants). We performed this single-pollutant + ozone analysis separately for each pollutant using the same data set as our primary analysis. As before, we estimated pollutant-property–specific CIs using a bootstrap procedure, based on resampling with replacement 500 times.

## Results

Table 2 summarizes statistics for cause-specific hospital admissions. For summary concentrations and percent distributions for the 65 pollutants included in our analyses, see Supplement Material, Table 1 (http://dx.doi.org/10.1289/ehp.1002646).

**Table 2:** Daily cause-specific hospital admissions, 1998–2006.

Percent distribution of daily admissions									Maximum daily admissions
Cause	5	25	50	75	95				
CVD		54		69		79		91		107		132
MI		1		2		3		5		7		12
CHF		13		18		21		26		33		47
IHD		13		18		22		26		32		40
Atrial fibrillation		11		16		19		23		30		39
Respiratory		25		32		38		44		56		76
COPD		11		16		20		24		32		43
ARI		0		0		1		2		3		10
Pneumonia		2		5		6		9		14		28
Causes were defined using ICD-9-CM codes. Data are for all years. Sample size is 729 days, with 271 in winter (November–March) and 458 in summer (April–October).												

**Figure 1:**
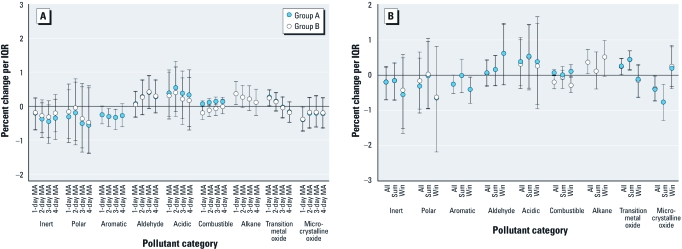
Percent change in CVD hospital admissions per IQR change in pollutant property by exposure window for all data (*A*) and by season (*B*) for 24-hr moving averages: two-pollutant property groupings (groups A and B). “Inert” does not include aromatics or alkanes; “polar” does not include aldehydes. Exposure windows include day of admission and 2-day, 3-day, and 4-day moving averages (MA) including day of admission. Abbreviations: Sum, summer season; Win, winter season.

*Hospital admissions for CVD disease.* Figure 1 shows the association between chemical properties (for groups A and B) and CVD hospital admissions for all year and by season [also see Supplemental Material, Table 2 (http://dx.doi.org/10.1289/ehp.1002646)]. For the 24-hr moving average, CVD-related hospital admissions increased significantly by 0.26% (group A; 95% CI, 0.02–0.48%) and 0.25% (group B; 95% CI, 0.00–0.47%) for an IQR increase in transition metals and by 0.37% (group B; 95% CI, 0.04–0.72%) for an IQR increase in alkanes, when we analyzed all data. Microcrystalline oxides were significantly and inversely associated with CVD hospital admissions (group A: –0.40; 95% CI, –0.74 to –0.03; group B: –0.39; 95% CI, –0.72 to –0.01). The percent change in the odds of CVD hospital admissions with an IQR change in transition metals and alkanes did not indicate associations when we averaged exposure over longer time periods, whereas corresponding percent changes for aromatics were similar across exposure windows (Figure 1A).

**Figure 2:**
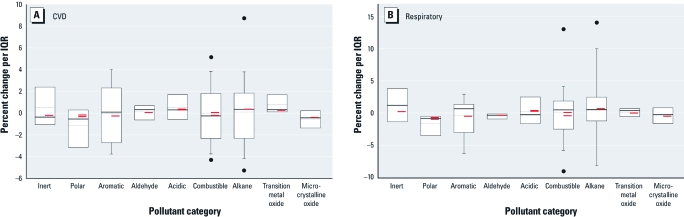
Percent change in CVD (*A*) and respiratory (*B*) hospital admissions per IQR change in each pollutant: results from first stage grouped by pollutant property (24-hr moving averages). “Inert” does not include aromatics and alkanes; “polar” does not include aldehydes. Solid circles indicate the 5th and 95th percentiles, whiskers the 10th and 90th percentiles, and boxes the 25th and 75th percentiles. The dotted (gray) line represents the median, while the solid line represents the mean. The red bars indicate percent change in CVD or respiratory hospital admissions for each pollutant property for 2-stage Group A and Group B models.

The association between CVD-related hospital admissions and pollutant properties was comparable under the alternative groupings (group A or B), except for combustible pollutants, for which the percent change was higher when we included aromatics compared with alkanes in the model. For transition metals, our two-stage model results were consistent with their respective individual pollutant-specific coefficients from the first-stage model, with all first-stage coefficients for the five pollutants classified as transition metals showing positive associations with CVD-related hospital admissions (Figure 2A). For alkanes, however, the distribution of relevant first-stage coefficients varied substantially and centered near zero (mean = 0.11); this result suggests that the positive association between alkanes and CVD hospitalization, when averaged over the 23 individual alkanes, was due to a subset of alkanes with positive and more precise coefficient estimates.

We found evidence that the relationship between CVD-related hospital admissions and pollutant properties differed by season (Figure 1B). In the summer, the odds of CVD-related hospital admissions increased significantly by 0.44% (95% CIs: group A, 0.16–0.68; group B, 0.14–0.69%) per IQR increase in 24-hr transition metals. Winter associations for transition metals were not significant (group A; –0.12; 95% CI, –0.62 to 0.31). Although not statistically significant in either season, associations with alkanes were positive in both seasons, with odds higher in the winter.

**Figure 3:**
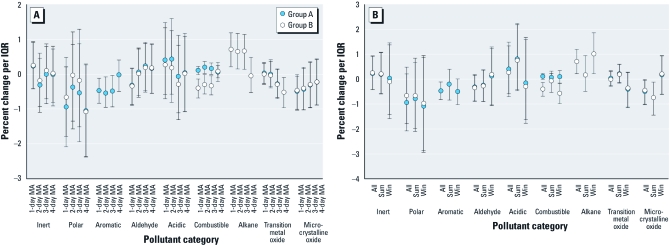
Percent change in respiratory hospital admissions per IQR change in pollutant property by exposure window for all data (*A*) and by season (*B*) for 24-hr moving averages: two-pollutant property groupings (groups A and B). “Inert” does not include aromatics and alkanes; “polar” does not include aldehydes. Exposure windows include day of admission and 2-day, 3-day, and 4-day moving averages (MA) including day of admission. Abbreviations: Sum, summer season; Win, winter season.

*Hospital admissions for respiratory disease.* An IQR increase in 24-hr alkanes was associated with a significant 0.71% increase (95% CI, 0.21–1.19%) in odds of respiratory-related hospital admissions [Figure 3A; see also Supplemental Material, Table 2 (http://dx.doi.org/10.1289/ehp.1002646)]. In contrast, odds of respiratory-related hospital admissions decreased significantly by 0.48% (95% CI, –0.83% to –0.12%) with an IQR increase in 24-hr aromatic levels [Figure 3A; see also Supplementary Materials, Table 2]. Results were relatively stable across exposure windows, with the exception of alkanes and aromatics, for which the 4-day moving averages showed null associations (Figure 3A). Results were relatively consistent when we analyzed data by season, although results suggested seasonal differences in odds of respiratory-related hospital admissions for alkanes and microcrystalline oxides (Figure 3B). For both alkanes and aromatics, findings from the second-stage model did not agree with the distributions of individual pollutant-specific coefficients from the first-stage model, which were variable with medians near zero (Figure 2).

**Figure 4:**
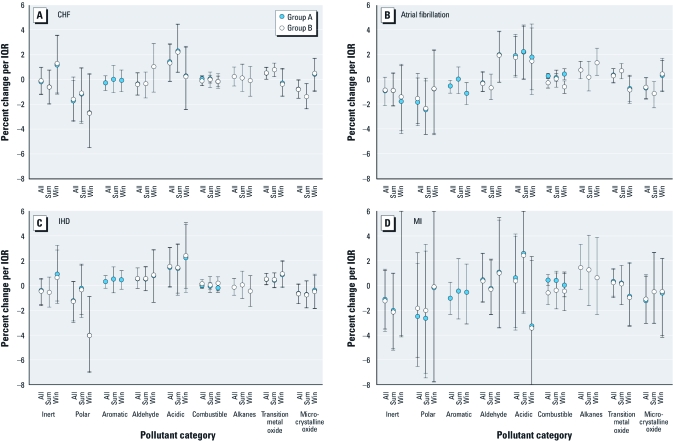
Relation of hospital admissions for CHF (*A*), IHD (*B*), atrial fibrillation (*C*), and MI (*D*) and 24-hr averaged pollutant properties, all year and by season: two-pollutant property groupings (groups A and B). “Inert” does not include aromatics and alkanes; “polar” does not include aldehydes. Abbreviations: Sum, summer season; Win, winter season.

*Admissions for specific cardiovascular-related causes.* We examined the relation between hospital admissions and pollutant categories for MI, atrial fibrillation, IHD, and CHF [Figure 4; see also Supplemental Material, Table 3 (http://dx.doi.org/10.1289/ehp.1002646)]. For all data, we again found transition metals to be significantly associated with increased hospital admissions for CHF and IHD (Figure 4; see also Supplemental Material, Table 3). The relation between transition metals and hospital admissions for IHD was positive and statistically significant across the year (group A, 0.50%; 95% CI, 0.05–0.98). When stratified by season, the association remained positive (group A: summer, 0.42%; 95% CI, –0.19 to 1.01; winter, 0.86%; 95% CI, –0.17 to 1.94; Figure 4). For hospital admissions for CHF, the relation with transition metals all year (group A; 0.54%; 95% CI, 0.04–0.99) and in the summer (0.78%; 95% CI, 0.24–1.32) was statistically significant and positive (Figure 4), whereas in the winter, associations were null with 95% CIs (group A; –0.27; 95% CI, –1.34 to 0.91) that did not overlap those in summer. Although not statistically significant, acidic pollutants also showed positive associations with hospital admissions for CHF (group A: 1.45%; 95% CI, –0.14 to 2.85) and IHD (group A: 1.46%; 95% CI, –0.14 to 3.03) for all year. By season, associations remained strongly positive. For example, in the summer, an IQR increase in 24-hr acidic pollutants was associated with a significant 2.32% increase (group A; 95% CI, 0.60–4.47%) in odds of CHF hospital admissions. Polar pollutants were significantly associated with decreased hospital admission for IHD in the winter and for CHF in all year and seasonal analyses. For atrial fibrillation, acidic pollutants, alkanes, and transition metals showed positive associations all year, although associations for transition metals were not significant (Figure 4; see also Supplemental Material, Table 3). In contrast, aromatics were significantly associated with decreased hospital admissions for atrial fibrillation. In the summer, we found significant positive associations for transition metals and acidic pollutants but inverse associations for microcrystalline oxides. In the winter, alkanes and aromatics were significantly associated with increased and decreased hospital admissions for atrial fibrillation, respectively. No pollutant category was significantly associated with hospital admissions for MI, possibly due to the small number of MI events.

**Table 3:** CVD hospital admission results by different first-stage models: 24-hr exposures on the day of admission [percent change (95% CI)].

Pollutant category	Multipollutant + ozone	Single pollutant + ozone
Inert		–0.203 (–0.701 to 0.222)		–0.270 (–0.703 to 0.046)
Polar		–0.316 (–1.075 to 0.484)		–0.332 (–1.141 to 0.568)
Aromatic		–0.256 (–0.520 to 0.001)		–0.141 (–0.324 to 0.069)
Aldehyde		0.064 (–0.307 to 0.438)		–0.314 (–1.135 to 0.316)
Acidic		0.381 (–0.299 to 1.065)		0.056 (–0.969 to 0.952)
Combustible		0.065 (–0.017 to 0.157)		–0.402 (–0.950 to 0.140)
Transition metal oxide		0.259 (0.023 to 0.479)		0.158 (–0.676 to 0.743)
Microcrystalline oxide		–0.401 (–0.737 to –0.032)		–0.534 (–1.126 to –0.078)

**Figure 5:**
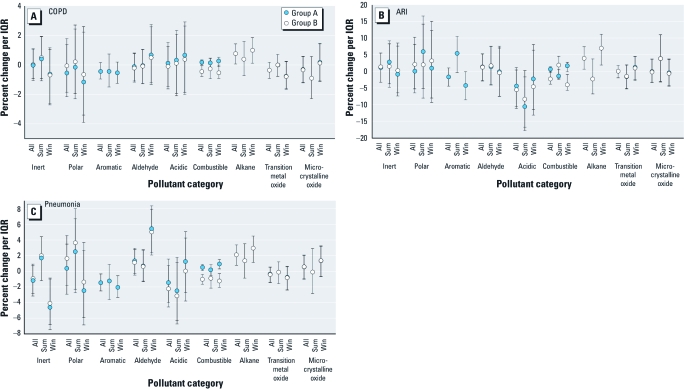
Relation of hospital admissions for COPD (*A*), ARI (*B*), and pneumonia (*C*) and 3-day averaged pollutant properties, all year and by season: two-pollutant property groupings (groups A and B). “Inert” does not include aromatics and alkanes; “polar” does not include aldehydes. Abbreviations: Sum, summer season; Win, winter season.

*Admissions for respiratory-related causes.* We examined the relation of chemical properties with hospital admissions for three specific respiratory-related causes: COPD, pneumonia, and ARI (Figure 5). We did so using the 3-day moving average given the findings of respiratory related visits and ozone from Peel et al. (2005). Results from these models show that alkanes were significantly positively associated with hospital admissions for pneumonia ARI, and COPD during the year and in winter, whereas aromatics tended to be inversely associated with these outcomes during the same time frame (Figure 5).

*Sensitivity analysis: single-pollutant + ozone and multipollutant + ozone models.* When we ran single-pollutant models adjusted for ozone and temperature (as opposed to multiple-pollutant models) in the first stage, the percent change in odds of hospital admissions for an IQR change in each of the pollutant properties generally fell within the 95% CI for the multivariate results (Table 3 lists group A results, which does not include alkanes). Of note, the relation of aldehydes and combustible pollutants with CVD hospital admissions from the single-pollutant models were in opposite direction compared with those from their multipollutant counterparts, resulting in the single-pollutant estimates being outside the 95% CI from the multipollutant results. In addition, the relation between transition metals and hospital admissions was no longer statistically significant in the single-pollutant models.

## Discussion

Using a hierarchical two-stage regression approach, we found consistent and significant associations between 24-hr transition metals and increased hospital admissions for CVD, CHF, and IHD and noted somewhat less consistent findings for atrial fibrillation and MI. We also found significant positive associations for alkanes and CVD hospital admissions. However, these associations were less stable than were those for transition metals, as shown by the individual pollutant-specific first-stage coefficients, which were highly variable and had a median value near zero. These findings suggest that positive overall associations may be due to a subset of alkanes. It is also possible that positive findings for alkanes may reflect the fact that this pollutant property was strongly correlated with aromatic pollutants, which together with microcrystalline oxides were generally inversely associated with CVD hospital admissions. Given their strong correlation, it was not possible to estimate associations of aromatics and alkanes with hospital admissions separately.

Observed associations with CVD hospital admissions suggested that pollutant impacts may vary by season. For example, the relationship between transition metals and hospital admissions for all CVD and for CHF and atrial fibrillation were significantly positive in the summer but were close to zero and null in the winter. Null wintertime associations may reflect the relatively small number of winter days included in our analysis, because prior studies have shown significant wintertime associations between CVD-related emergency department visits and sources such as traffic and metal processing that include transition metals (Sarnat et al. 2008). Stronger associations in the summer compared with winter are contrary to previous observations of a protective impact of air conditioning (Janssen et al. 2002; Sarnat et al. 2000). Alkanes showed the opposite seasonal relationship, with significant positive associations for winter but not summer.

The two pollutant property groupings (A and B) produced similar results, although coefficients for combustible pollutants were generally higher when we included aromatics compared with alkanes in second-stage models. Correspondingly, when we used single-pollutant models instead of multiple-pollutant models in the first stage, results were generally similar. Single-pollutant first-stage model coefficients for aldehydes and combustible pollutants, however, were of the opposite sign and outside the 95% CI of our multiple-pollutant results but remained insignificant.

Our findings of significant associations for transition metals (e.g., titanium, zinc, manganese, iron, and chromium) are biologically plausible. Transition metals originate from diverse sources and have, among other qualities, positive oxidation states. These positive states suggest that transition metals may react with oxygen in the human body to yield highly reactive oxygen species (ROS), including hydroxyl radicals. Once formed, ROS can cause harm by disturbing mitochondrial function, reducing nicotinamide adenine dinucleotide phosphate-oxidase, and activating inflammatory cells capable of generating ROS, reactive nitrogen species, and oxidative DNA damage (Dhalla et al. 2000; Rison et al. 2005). Correspondingly, several epidemiological studies have found associations between transition metals, most notably vanadium, nickel, and chromium, and increased hospital admissions (Bell et al. 2009; Zanobetti et al. 2009), mortality (Dominici et al. 2007; Lippmann et al. 2006), and intermediate impacts such as inflammation, autonomic dysfunction, and oxidative stress (Park et al. 2006; Urch et al. 2004).

Although health studies of associations between transition metals as a group and adverse health impacts have not been performed, Sarnat et al. (2008) linked transition metals indirectly to increased emergency department visits in Atlanta. In this study, CVD emergency department visits were positively associated with factors consistent with transportation, metal processing, and biomass burning. For each of these factors, transition metals explained a high percentage of variation. IQR increases in these sources were associated with relative risk estimates ranging between 1.013 for metal processing and 1.033 for biomass burning. For the transition metal zinc alone, Sarnat et al. (2008) estimated a relative risk of 1.013 (95% CI, 1.005–1.022). Findings from this study qualitatively lend support for our findings.

Less is known about the health impacts of alkanes. Alkanes, such as methane and 2,2-dimethylbutane, are hydrocarbons that consist of only carbon and hydrogen linked together exclusively by single bonds. Alkanes are not reactive and have little known biological activity. Aromatics include chemicals, such as benzene and toluene, with a set of ringed, covalently bound atoms that have alternating single and double bonds and a coplanar structure and that follow Hückel’s rule (e.g., having 4*n* + 2 electrons in the delocalized *p*-orbital cloud). Although aromatics are also stable compounds, they are associated with serious health effects. Benzene, for example, adversely affects the nervous and hematopoietic system and is a known human carcinogen, and toluene has been shown to damage kidneys, the nervous system, liver, brain, and heart (U.S. EPA 2010). The known toxicity of benzene, toluene, and other aromatics suggests that our findings of inverse associations for aromatics and positive associations for alkanes may reflect their strong correlation with one another.

Our study is limited by our use of air pollution measurements from a single stationary ambient monitoring site as our exposure measure, which may cause exposure misclassification. The extent of this misclassification likely differs by pollutant, depending on its spatial profile and relation to personal exposures. Resulting exposure misclassification, however, will likely bias our effect estimates toward the null (Zeger et al. 2000). The relative strength of the associations with the pollutant categories, however, should be interpreted in light of these limitations.

## Conclusion

Our findings provide the first evidence suggesting an association between transition metals and other pollutant properties and hospital admissions and demonstrate the utility of a new approach to assess the health impacts of air pollutant mixtures. This approach should be replicated in different cities, especially those with observed strong health–exposure associations for the criteria pollutants, and using different health outcomes and pollutant properties (e.g., particle size) to assess the robustness of our findings and method.

## Supplemental Material

(104 KB) PDF
