# Supplementary material for: Chemical Properties of Air Pollutants and Cause-Specific Hospital Admissions among the Elderly in Atlanta, Georgia
Source: Environ Health Perspect. 2011 Jun 27;119(10):1421–8. doi: 10.1289/ehp.1002646 (PMC3230427; doi:10.1289/ehp.1002646)
Supplement: (104 KB) PDF [file ehp.1002646.s001.pdf]

Supplemental Information

Chemical Properties of Air Pollutants and Cause-Specific Hospital Admissions among the Elderly in Atlanta, GA

Helen H. Suh[1,2], Antonella Zanobetti[2], Joel Schwartz[2], and Brent Coull[3]

Affiliations: [1]Environmental Health Program, NORC at the University of Chicago, [2]Department of Environmental Health, Harvard School of Public Health, Boston, MA [3]Department of Biostatistics, Harvard School of Public Health, Boston, MA

Corresponding Author: Helen H. Suh

Environmental Health Program

NORC at the University of Chicago

Boston, MA 02215

Suh-helen@norc.org

Table of Contents

Supplemental Material, Table 1: Summary of Pollutant Concentrations…..…………….…..…3

Supplemental Material, Table 2: Association of Pollutant Properties and Hospital Admissions for 24-h Exposures on Day of Admission……………………………………………………6

Supplemental Material, Table 3. Association of Pollutant Properties and Hospital Admissions for CVD Causes for 24-h Exposures on the Day of Admission………………………………..,7

Supplemental Material, Table 1. Summary of Pollutant Concentrations

Variable	Mean	Std Dev	Min	10%	25%	50%	75%	IQR	IQR/ median
Acetic Acid (ppb)	7.21	7.12	0.05	0.50	1.80	5.25	10.55	8.75	1.67
Acetylene (ppb)	6.16	6.30	0.20	1.80	2.65	4.20	6.80	4.15	0.99
Arsenic (ng/m^3)	1.42	1.44	0.50	0.51	0.51	0.58	2.00	1.50	2.59
Benzaldehyde (ppb)	2.47	2.17	0.05	0.90	1.30	2.00	2.90	1.60	0.80
Benzene (ppb)	3.81	3.26	0.70	1.60	2.00	2.70	4.30	2.30	0.85
Bromine (ng/m^3)	3.51	3.08	0.26	1.02	1.82	2.82	4.36	2.54	0.90
i-Butane (ppb)	3.93	4.08	0.30	1.10	1.60	2.60	4.70	3.10	1.19
n-Butane (ppb)	10.37	13.14	1.00	2.30	3.50	6.10	11.25	7.75	1.27
2-Butanone (ppb)	2.28	1.55	0.05	0.70	1.20	2.00	3.00	1.80	0.90
1-Butene (ppb)	0.54	0.64	0.05	0.10	0.25	0.40	0.60	0.35	0.88
i-Butene (ppb)	0.85	0.98	0.05	0.20	0.30	0.50	1.00	0.70	1.40
CaO (ug/m^3)	0.06	0.05	0.00	0.02	0.03	0.05	0.08	0.05	0.95
CO (ppb)	472.44	296.18	155.46	233.49	291.51	383.55	537.51	245.99	0.64
Copper (ng/m^3)	3.55	11.29	0.61	0.62	0.62	0.63	2.98	2.36	3.73
Cyclopentane (ppb)	0.48	0.47	0.05	0.10	0.20	0.30	0.50	0.30	1.00
n-Decane (ppb)	1.25	1.35	0.05	0.40	0.50	0.90	1.40	0.90	1.00
Dew Point	11.13	8.53	-13.10	-1.66	4.83	13.38	18.55	13.72	1.03
2,2-Dimethylbutane (ppb)	0.94	0.88	0.05	0.20	0.40	0.60	1.20	0.80	1.33
2,3-Dimethylbutane (ppb)	1.11	1.17	0.05	0.30	0.50	0.70	1.30	0.80	1.14
2,3-Dimethylpentane	1.04	1.08	0.05	0.30	0.40	0.70	1.30	0.90	1.29
2,4-Dimethylpentane	0.72	0.79	0.05	0.20	0.30	0.40	0.85	0.55	1.38
Elemental Carbon	1.53	0.97	0.17	0.59	0.85	1.29	1.92	1.07	0.83
3-Ethylhexane (ppb)	0.60	0.63	0.05	0.20	0.30	0.40	0.70	0.40	1.00
Ethylbenzene (ppb)	2.47	2.46	0.20	0.80	1.10	1.60	2.80	1.70	1.06
Ethane (ppb)	9.10	7.93	2.00	3.70	5.00	7.00	10.15	5.15	0.74
Ethylene (ppb)	5.58	5.33	0.30	1.70	2.50	3.80	6.40	3.90	1.03
m-Ethyltoluene (ppb)	1.12	1.32	0.05	0.30	0.50	0.80	1.30	0.80	1.00
p-Ethyltoluene (ppb)	2.29	2.58	0.05	0.70	1.00	1.60	2.70	1.70	1.06
Fe_2O_3 (ug/m^3)	0.11	0.08	0.01	0.04	0.06	0.08	0.14	0.08	0.92
Heptanal (ppb)	0.86	0.57	0.05	0.30	0.50	0.70	1.10	0.60	0.86

Supplemental Table 1 (continued)

Variable	Mean	Std Dev	Min	10%	25%	50%	75%	IQR	IQR/ median
n-Heptane (ppb)	1.48	1.49	0.05	0.50	0.70	1.00	1.60	0.90	0.90
Hexanal (ppb)	1.04	0.72	0.05	0.40	0.60	0.90	1.30	0.70	0.78
n-Hexane (ppb)	2.55	2.64	0.40	0.90	1.10	1.60	2.85	1.75	1.09
Nitric Acid	1.16	1.08	0.06	0.26	0.39	0.77	1.58	1.19	1.55
K_2O (ug/m^3)	0.07	0.07	0.01	0.03	0.04	0.06	0.08	0.05	0.79
Lead (ng/m^3)	5.19	9.40	1.16	1.17	1.17	2.49	5.78	4.62	1.86
Manganese (ng/m^3)	1.52	1.20	0.40	0.40	0.40	1.26	2.16	1.76	1.40
Methylcyclohexane	0.92	1.14	0.05	0.20	0.30	0.50	1.00	0.70	1.40
Methylcyclopentane	1.43	1.49	0.05	0.50	0.60	0.90	1.60	1.00	1.11
2-Methylhexane (ppb)	1.39	1.36	0.05	0.40	0.60	0.90	1.60	1.00	1.11
3-Methylhexane (ppb)	1.80	1.56	0.05	0.60	0.90	1.30	2.20	1.30	1.00
2-Methylheptane (ppb)	0.56	0.55	0.05	0.20	0.30	0.40	0.70	0.40	1.00
2-Methylpentane (ppb)	3.60	3.79	0.50	1.10	1.50	2.20	4.10	2.60	1.18
3-Methylpentane (ppb)	2.14	2.31	0.05	0.60	0.90	1.30	2.40	1.50	1.15
m-Xylene, p-Xylene	7.45	8.41	0.70	2.00	2.90	4.55	8.50	5.60	1.23
Nitrogen Dioxide (ppb)	19.55	8.24	5.02	9.89	13.77	18.61	24.30	10.54	0.57
Nonane (ppb)	0.90	0.92	0.05	0.30	0.40	0.60	1.10	0.70	1.17
O_3 (ppb)	25.75	13.45	0.98	9.81	14.97	23.69	35.18	20.21	0.85
OC (ug/m^3)	4.21	2.21	0.39	2.08	2.69	3.76	5.12	2.44	0.65
Octanal (ppb)	1.59	1.17	0.05	0.40	0.90	1.30	2.10	1.20	0.92
n-Octane (ppb)	0.72	0.68	0.05	0.20	0.30	0.50	0.80	0.50	1.00
i-Pentane (ppb)	12.20	13.31	1.60	3.60	5.00	7.50	13.90	8.90	1.19
n-Pentane (ppb)	5.33	5.45	0.30	1.80	2.40	3.50	6.05	3.65	1.04
n-Propylbenzene (ppb)	0.69	0.78	0.05	0.20	0.30	0.50	0.80	0.50	1.00
Propane (ppb)	18.12	22.89	1.50	4.60	6.60	10.50	20.10	13.50	1.29
Propene (ppb)	2.85	3.28	0.40	0.80	1.10	1.65	3.30	2.20	1.33
Sulfur (ng/m^3)	1675.95	1034.75	274.29	666.17	934.32	1382.83	2178.04	1243.72	0.90
Selenium (ng/m^3)	1.32	1.21	0.35	0.35	0.35	1.04	1.86	1.51	1.46
SiO_2 (ug/m^3)	0.20	0.21	0.01	0.05	0.08	0.15	0.25	0.17	1.14
SO_2 (ppb)	5.13	4.27	0.05	1.20	2.06	3.75	7.05	5.00	1.33
Temperature (oC)	18.31	7.48	-4.44	6.67	12.77	19.16	24.44	11.67	0.61
Titanium (ng/m^3)	3.85	3.79	2.13	2.14	2.14	2.14	4.43	2.29	1.07

Supplemental Table 1 (continued)

Variable	Mean	Std Dev	Min	10%	25%	50%	75%	IQR	IQR/ median
1,2,4-Trimethy-lbenzene, sec-Butylbenzene (ppb)	3.13	3.44	0.05	0.90	1.30	2.00	3.60	2.30	1.15
1,3,5-Trimethylbenzene (ppb)	1.29	1.57	0.05	0.30	0.50	0.90	1.50	1.00	1.11
Toluene (ppb)	12.49	11.79	0.50	4.20	5.65	8.45	14.40	8.75	1.04
2,2,4-Trimethylpentane (ppb)	3.98	4.27	0.05	1.10	1.50	2.50	4.70	3.20	1.28
2,3,4-Trimethylpentane (ppb)	1.28	1.42	0.05	0.30	0.50	0.80	1.50	1.00	1.25
o-Xylene (ppb)	2.87	3.00	0.30	0.90	1.20	1.80	3.40	2.20	1.22
Zinc (ng/m^3)	11.70	9.25	0.39	3.61	6.01	9.40	14.60	8.59	0.91

Our analysis included data for a total of 729 days, which included valid measurements for all pollutants of interest. Data from 1998-2006.

Supplemental Material, Table 2. Association of Pollutant Properties and Hospital Admissions for 24-h Exposures on Day of Admission

Pollutant Property	Group A			Group B		
	%change[a]	2.5%	97.5%	%change[a]	2.5%	97.5%
CVD						
Inert[b]	-0.203	-0.701	0.222	-0.193	-0.696	0.245
Polar[c]	-0.316	-1.075	0.484	-0.164	-0.961	0.652
Aromatic	-0.256	-0.520	0.001			
Aldehyde	0.064	-0.307	0.438	0.053	-0.329	0.421
Acidic	0.381	-0.299	1.065	0.312	-0.376	1.001
Combustible	0.065	-0.017	0.157	-0.201	-0.388	-0.012
Alkanes				0.367	0.041	0.723
Transition Metal	0.259	0.023	0.479	0.246	0.003	0.470
Microcrystalline Oxide	-0.401	-0.737	-0.032	-0.385	-0.724	-0.009
Respiratory						
Inert[a]	0.225	-0.422	0.905	0.249	-0.418	0.928
Polar[b]	-0.944	-2.086	0.205	-0.666	-1.790	0.480
Aromatic	-0.475	-0.832	-0.117			
Aldehyde	-0.321	-0.877	0.174	-0.341	-0.892	0.154
Acidic	0.402	-0.590	1.479	0.268	-0.709	1.331
Combustible	0.108	-0.011	0.224	-0.401	-0.684	-0.140
Alkanes				0.707	0.209	1.187
Transition Metal	0.026	-0.263	0.334	0.003	-0.288	0.298
Microcrystalline Oxide	-0.498	-1.030	-0.064	-0.467	-1.006	-0.019

[a] "%change" expressed per IQR for pollutants in their respective categories
[b] "Inert" does not include pollutants that are aromatic or alkanes.
[c] "Polar" does not include pollutants that are aldehydes.

Supplemental Material, Table 3. Association of Pollutant Properties and Hospital Admissions for CVD Causes for 24-h Exposures on the Day of Admission

Pollutant Property	Group A			Group B		
	%change	2.5%	97.5%	%change	2.5%	97.5%
IHD						
Inert	-0.399	-1.559	0.576	-0.468	-1.617	0.489
Polar	-1.237	-2.854	0.318	-1.307	-2.985	0.305
Aromatic	0.306	-0.231	0.786			
Aldehyde	0.539	-0.254	1.389	0.558	-0.241	1.406
Acidic	1.457	-0.142	3.032	1.519	-0.101	3.060
Combustible	-0.032	-0.197	0.157	0.130	-0.253	0.480
Alkanes				-0.142	-0.792	0.570
Transition Metal	0.496	0.046	0.975	0.517	0.070	0.979
Microcrystalline Oxide	-0.649	-1.466	0.121	-0.665	-1.474	0.090
CHF						
Inert	-0.216	-1.223	0.971	-0.177	-1.182	0.987
Polar	-1.765	-3.392	-0.081	-1.664	-3.294	0.184
Aromatic	-0.269	-0.892	0.287			
Aldehyde	-0.301	-1.251	0.529	-0.317	-1.253	0.535
Acidic	1.453	-0.142	2.846	1.389	-0.168	2.867
Combustible	0.067	-0.139	0.297	-0.137	-0.505	0.270
Alkanes				0.241	-0.526	0.998
Transition Metal	0.535	0.037	0.988	0.520	0.021	0.965
Microcrystalline Oxide	-0.808	-1.509	-0.046	-0.793	-1.502	-0.032
MI						
Inert	-1.143	-3.497	1.318	-1.233	-3.695	1.220
Polar	-2.498	-6.507	1.784	-1.855	-5.777	2.646
Aromatic	-1.034	-2.316	0.252			
Aldehyde	0.428	-1.279	2.606	0.359	-1.352	2.571
Acidic	0.597	-3.390	4.160	0.361	-3.592	3.968
Combustible	0.433	-0.005	0.890	-0.595	-1.536	0.384
Alkanes				1.427	-0.310	3.308
Transition Metal	0.312	-0.854	1.362	0.244	-0.964	1.327
Microcrystalline Oxide	-1.205	-3.036	0.756	-1.120	-3.031	0.872
Atrial Fibrillation						
Inert	-0.928	-2.117	0.162	-0.899	-2.110	0.148
Polar	-1.858	-3.757	0.087	-1.567	-3.570	0.463
Aromatic	-0.541	-1.123	-0.087			
Aldehyde	-0.304	-0.955	0.610	-0.329	-1.004	0.572
Acidic	1.893	0.288	3.612	1.751	0.172	3.451
Combustible	0.264	0.074	0.458	-0.278	-0.700	0.081
Alkanes				0.736	0.047	1.446
Transition Metal	0.339	-0.249	0.848	0.313	-0.290	0.837
Microcrystalline Oxide	-0.706	-1.593	0.099	-0.673	-1.548	0.142
